# Biomimetic, ultrathin and elastic hydrogels regulate human neutrophil extravasation across endothelial-pericyte bilayers

**DOI:** 10.1371/journal.pone.0171386

**Published:** 2017-02-24

**Authors:** Holly M. Lauridsen, Anjelica L. Gonzalez

**Affiliations:** Department of Biomedical Engineering, Yale University, New Haven, CT, United States of America; University of Michigan, UNITED STATES

## Abstract

The vascular basement membrane—a thin, elastic layer of extracellular matrix separating and encasing vascular cells—provides biological and mechanical cues to endothelial cells, pericytes, and migrating leukocytes. In contrast, experimental scaffolds typically used to replicate basement membranes are stiff and bio-inert. Here, we present thin, porated polyethylene glycol hydrogels to replicate human vascular basement membranes. Like commercial transwells, our hydrogels are approximately 10μm thick, but like basement membranes, the hydrogels presented here are elastic (E: 50-80kPa) and contain a dense network of small pores. Moreover, the inclusion of bioactive domains introduces receptor-mediated biochemical signaling. We compare elastic hydrogels to common culture substrates (E: >2GPa) for human endothelial cell and pericyte monolayers and bilayers to replicate postcapillary venules *in vitro*. Our data demonstrate that substrate elasticity facilitates differences in vascular phenotype, supporting expression of vascular markers that are increasingly replicative of venules. Endothelial cells differentially express vascular markers, like EphB4, and leukocyte adhesion molecules, such as ICAM-1, with decreased mechanical stiffness. With porated PEG hydrogels we demonstrate the ability to evaluate and observe leukocyte recruitment across endothelial cell and pericyte monolayers and bilayers, reporting that basement membrane scaffolds can significantly alter the rate of vascular migration in experimental systems. Overall, this study demonstrates the creation and utility of a new and accessible method to recapture the mechanical and biological complexity of human basement membranes *in vitro*.

## Introduction

The vascular basement membrane (BM) is a highly specialized layer of extracellular matrix in the sub-endothelial space. The BM plays two critical roles: it provides a structural scaffold for resident vascular cells and provides localized mechanical and biological signals to resident and migrating cells. In its role as a scaffold, endothelial cells (ECs) adhere to the luminal face of the BM, whereas perivascular pericytes (PCs) are located in the abluminal portion of the vessel. The BM ranges in thickness from 50 to 300nm; ECs and PCs utilize the pores or voids within the BM to make contact with one another [1]. Collagen IV, laminin-8, and laminin-10 are three of the primary extracellular matrix (ECM) constituents of the venular BM, with proteoglycans like nidogen and perlecan, and growth factors such as basic fibroblast growth factor and platelet derived growth factor representing a smaller portion of the matrix [2–4]. Collagen and laminin are also largely responsible for controlling the mechanical properties of the BM [5], as evidenced by the correlation between vascular stiffening and increased collagen and laminin observed in the vasculature of in diabetic tissues [6]. Thus, dynamic changes in the composition of the BM that are associated with aging or disease result in changes to the mechanical properties of the vascular wall [5].

Resident ECs and PCs, and transient inflammatory cells have the capacity to sense biological and mechanical alterations within the vascular wall and consequently alter their behavior [7]. *In vivo*, local mechanical cues, including substrate stiffness, regulate EC phenotype by altering the production of glycocalyx [8], expression of arterial or venous markers [9], or organization of the cytoskeleton [10]. Microenvironmental effects on PC phenotype are less well understood, though studies indicate that PC survival is associated with changes in the BM (e.g. diabetic remodeling of the retina) [6,11]. BM mechanics also determine the behavior of migrating cells, such as leukocytes during inflammation. Neutrophils rapidly probe their surroundings during migration and alter their movement in response to mechanical cues [7,12,13]. Our previous research demonstrates that both local and bulk mechanical properties of ECM-like substrates modulate neutrophil integrin-independent chemotaxis [12].

Rigid and bio-inert substrates such as tissue culture polystyrene, polycarbonate transwells, and glass are common and easy methods for cell culturing and single cell assays [14,15]. These traditional substrates, however, fail to fully replicate the biological and mechanical complexity of the BM [16]. The stiffness of human BMs can range from 2-80kPa [17], a marked contrast to the Young’s modulus of commonly used culture substrates: the Young’s moduli of polystyrene, glass, and polycarbonate are 3.7, 35–75, and 2.5GPa, respectively [18–20]. Here, we present a method for creating thin, porated PEG hydrogels that provide a biomimetic alternative to traditional culture substrates for modeling human BMs. These porated PEG hydrogels replicate the mechanically pliable environment of the BM and contain a dense network of small pores to mimic the BM architecture. We demonstrate the use of these hydrogels for mono- and co-culture of vascular cells, recreating the complex construct of the human microvasculature. The use of elastic scaffolds for vascular cell culture restores *in vivo* characteristics of microvascular cells, facilitating the expression of venular EC markers. Moreover, we validate that porated PEG hydrogels can be used for leukocyte recruitment assays, providing a method to evaluate the frequently ignored interactions between migrating leukocytes and the vascular ECM. We demonstrate that both the elasticity and bioactivity provided by a bioactive scaffold can alter the efficiency of neutrophil migration relative to migration observed on stiff and bio-inert alternatives. Overall, the ultrathin porated PEG hydrogels represent an evolution in *in vitro* models of human BM, that here, recapitulate the mechanical and biological signals of human microvasculature.

## Materials and methods

### Ethics statement

This study, and the use and attainment of human cells were approved by Yale University and its Human Investigation Committee (HIC) of the Institutional Review Board (IRB) as part of the Human Research Protection Program. This research was conducted in accordance with protocols in line with the standards set by the Helsinki Declaration. All advertisements for volunteers were approved by the Yale University HIC IRB protocol # 0902004786 and written informed consent was obtained from all human volunteers prior to blood collection.

### Zinc oxide micro-needles and slide coatings

Zinc oxide micro-needles were formed according to the method described in McBride et al. [21]. In brief, 250mL of 0.04M zinc nitrate hexahydrate (Sigma Aldrich, St. Louis MO) was mixed with 150mL of 1M sodium hydroxide for 2 hours at room temperature. The solution was heated to its reflux temperature of 55–60°C and maintained at that temperature for 7–24 hours. The resulting zinc oxide microparticles were filtered out of solution and dried overnight; formation of micro-needles was confirmed via scanning electron microscopy on a Hitachi SU-70 scanning electron microscope at 10kV. Zinc oxide sacrificial layers were formed on plain microscope slides; slides were rinsed with 70% ethanol, air-dried, and coated with a 0.048M zinc acetate (Sigma-Aldrich) in methanol. Coated slides were heated to 215°C for a minimum of 14 minutes on a hot plate and UV sterilized prior to use.

### Hydrogel production

10kDa and 20kDa polyethylene glycol diacrylate (PEG-DA) was synthesized as previously described [12]. In brief, PEG (Polysciences Inc., Warrington PA) was reacted with acryloyl chloride (Sigma-Aldrich) and triethylamine (Sigma-Aldrich) overnight in anhydrous dichloromethane (American Bioanalytical, Natick, MA). Potassium carbonate was added to induce phase-separation. The organic phase was collected, dried with magnesium sulfate, filtered, and precipitated with diethyl ether. PEG-DA was dialyzed and lyophilized prior to use. PEG conjugated to the tripeptide Arg-Gly-Asp (RGD; Lifetein) was also produced as previously described [12]. Briefly, arginylglycylaspartic acid was conjugated to acryloyl–PEG–N-hydroxysuccinimide (PEG–NHS, MW 3500 Da, JenKem, Allen TX) in sodium bicarbonate (pH 8.5) in a 1:1 molar ratio. Conjugated PEG was dialyzed and lyophilized prior to use. Here, hydrogels were created by suspending 37mg/mL of zinc micro-needles in 10% fetal bovine serum in water. The micro-needle solution was vortexed and briefly centrifuged to remove any large zinc oxide aggregates. PEG hydrogels containing RGD were made by dissolving 50mgl/mL PEG-DA, 10mg/mL PEG-RGD, and 8% crosslinker (300mg/mL acetophenone/N-vinyl pyrrilidone) in the micro-needle solution. 20μL of the final solution was sandwiched between zinc oxide coated slides and cross-linked under a UV light for 15–30 min. After crosslinking, microscope slides were carefully separated leaving the hydrogel attached on one slide. Released hydrogels were removed from the slide with a 1M hydrochloric acid rinse and gentle agitation. The hydrogel was thoroughly rinsed with PBS and moved to a six-well plate with PBS using a punched silicone membrane (Grace BioLabs, Eugene, OR). Gels were UV sterilized for one hour and repeatedly rinsed with PBS to remove residual acid. Hydrogels were transferred to new plates and rinsed with PC culture media containing phenol red to verify a neutral pH.

### Mechanical testing

The Young’s modulus of the hydrogel was determined via a modified ball drop method, as previously described [12,22]. In summary, hydrogels were secured on open based platforms. Steel balls of known dimensions were added to the hydrogels to induce elastic deformations; deformations were captured on camera and measured with ImageJ. Given the dimensions of the hydrogel and the ball, the Young’s Modulus was calculated. The thickness of each gel was determined via optical coherence tomography (OCT; Callisto Model, Thorlabs, Newton, NJ).

### Cell culture and substrate seeding

Human umbilical vein ECs were isolated according to the procedures described in Gimbrone et al. [23]. Harvested ECs were subsequently cultured in 0.1% gelatin-coated tissue culture flasks at 37°C with 5%CO_2_. ECs were sustained with complete culture media (M199 medium (Gibco, Grand Island, NY), 20% FBS (Hyclone Laboratories, Inc., Logan, UT), 1% penicillin-streptomycin (Gibco)) supplemented with 10μg/ml endothelial cell growth supplement (Collaborative Biomedical Products, Bedford, MA). All ECs were used between passage 2 and 5. Human placental PCs were isolated by explant-outgrowth methods from freshly isolated microvessels obtained from donated and anonymized human placentas; the methods of Maier et al. were followed [24]. PCs were cultured in T75s flasks with complete culture media. All PCs were used between passage 4 and 9. Confluent T75 flasks were trypsinized with 0.25% Trypsin EDTA (Gibco) and seeded onto appropriate substrates. To seed the substrates, cell suspensions were added drop-wise to gels to form a meniscus and incubated for ~3 hours before gently filling the well with media. For EC/PC bilayers, the ECs were seeded first and allowed to adhere for 12–24 hours. A second silicone membrane was used to sandwich the hydrogel and the gel was carefully inverted into a clean well. PCs were then seeded on the opposing surface as previously described. For TW controls, 6.5mm diameter TWs with 3 micron pores (Corning, Inc.) were coated with a 20% RGDS solution and air-dried. EC and PC monolayers or EC/PC bilayers were then seeded as previously described [14].

### Neutrophil isolation, adhesion, and capture assays

Peripheral blood neutrophils were isolated from healthy human volunteers in accordance with the Yale Internal Review Board protocol as previously described [14]. In summary, human neutrophils were isolated from the peripheral blood of healthy volunteers into syringes containing citrate phosphate dextrose and dextran. Blood was allowed to settle and plasma was collected. Neutrophils were isolated using a Histopaque gradient, and subsequently rinsed in PBS. Neutrophils were suspended at their final concentration of 6x10^6^ cells/mL in a PBS-glucose solution containing magnesium and calcium.

For neutrophil capture studies, a punched silicone sheet was added to the top of the hydrogel for stabilization, allowing cell solution to be applied directly to the apical surface of the hydrogel. The hydrogel was then suspended above an agarose (2%)-coated well in a 24 well plate. Wells contained complete cell culture media; for neutrophil chemotaxis assays, the well media was supplemented with 100ng/mL interleukin-8 (IL-8, eBioscience, San Diego, CA) to establish a chemotactic gradient. Cells were allowed to transmigrate for 1 hour. After 1 hour, the media in the basal chamber was collected and neutrophils that successfully transmigrated were counted. TWs and hydrogels were also collected, fixed, and stained with DAPI. Captured neutrophils were identified by their multi-lobed nuclei.

### Fluorescent imaging and flow cytometry

Cells were fixed with 4% paraformaldehyde and blocked with 2% bovine serum albumin (Santa Cruz Biotechnology, Inc.). Cells stained for intercellular adhesion molecule 1 (R&D Systems), vascular cell adhesion molecule 1 (Santa Cruz Biotechnology, Inc.), E-selectin (R&D Systems), NG2 (Santa Cruz Biotechnology, Inc.), PDGFRβ (Santa Cruz Biotechnology, Inc.), VE-Cadherin (eBioscience), and actin (Sigma-Aldrich) were not permeabilized. Cells stained for Connexin40 (Santa Cruz Biotechnology, Inc.), Connexin43 (Santa Cruz Biotechnology, Inc.), and EphB4 (R&D Systems were permeabilized with 0.1% Triton X-100 in the blocking solution. All primary antibodies were labeled with FITC or TRITC via appropriate secondary antibodies. Quantification of fluorescent marker expression was done via flow cytometry on an LSRII flow cytometer (BD Biosciences); analysis was completed using BD Diva software and Flowjo (Flowjo Enterprises, Ashland, OR). Samples prepared for fluorescent imaging were also labeled with DAPI using DAPI dye or Vectashield mounting medium with DAPI (Vector Labs, Burlingame, CA). Fluorescent imaging of cultured cells was completed on a Zeiss Axiovert microscope or a LSM 510 laser scanning confocal microscope (Zeiss, Thornwood, NY, USA). H&E stained sections of human skin biopsies were provided by the Yale School of Medicine’s Dermatology Department. Stained sections were imaged on a TiE inverted Nikon confocal microscope fit with a Yokogawa CSU-W1 spinning disk.

### Statistics

All statistical analysis was completed using GraphPad Prism (GraphPad Prism, San Diego, CA). Significance was determined using either a one-way or two-way ANOVAs with a Tukey post-hoc test or a two-tailed unpaired t-test as appropriate. Levels of significance are denoted as * or # p<0.05, **p<0.01, and ***p<0.001. All conditions have a sample size of three or more samples; outliers beyond two standard deviations from the mean were excluded from analysis.

## Results

### Porated PEG hydrogels provide an elastic alternative to rigid substrates

**Fig 1:**
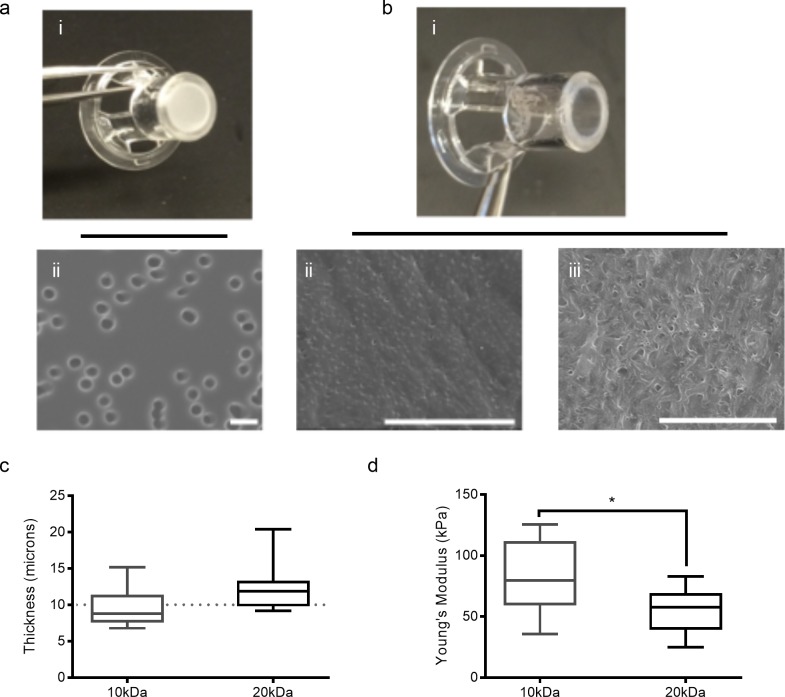
Zinc oxide micro-needles introduce pores into PEG hydrogels. (a-b) Images of commercial TWs (a-i) and porated PEG hydrogels in TW casing (b-i). SEM micrographs of TWs (a-ii), 10kDa (b-ii), and 20kDa (b-iii) porated PEG hydrogels; scale bars: 5 microns. (c) Hydrogel thickness as determined by OCT. Whiskers denote minimum and maximum value; there is no statistical difference between 10kDa and 20kDa hydrogels. (d) Young’s modulus of 10kDa and 20kDa porated PEG hydrogels. Whiskers denote minimum and maximum value; *p<0.05.

In place of the traditional polycarbonate transwell (TW) (Fig 1A), Zinc oxide needle-like microparticles (“micro-needles) were included in a PEG hydrogel precursor solution, cross-linked, and subsequently dissolved to create a thin and transparent substrate (Fig 1B) containing a randomly distributed pore network. Pore size and density were quantified, revealing that, in contrast to commercially available transwells (mean pore diameter 2.52 microns; mean pore density of 2.9x10^4^ pores/mm^2^ ± 2.3 x10^3^ pores/mm^2^ (s.e.m.)) porated hydrogels contained a higher density of smaller pores, increasingly replicative of human basement membrane. 10kDa hydrogels contained a mean pore diameter of 0.19μm and pore density of 8.8x10^5^ pores/mm^2^ ± 7.0 x10^5^ pores/mm^2^ (s.e.m.). Similarly, the mean pore diameter of the 20kDa hydrogels was 0.16μm and a pore density of 3.5x10^6^ pores/mm^2^ ± 2.2 x10^6^ pores/mm^2^ (s.e.m.).

TWs are typically polycarbonate sheets with a thickness of 10 microns, providing sufficient support and space for cell-cell contacts between cross-membrane cultured bilayers. Using optical coherence tomography (OCT), we evaluated the thickness of the PEG hydrogels in their hydrated state (Fig 1C). Both the 10kDa and 20kDa hydrogels had a mean thickness comparable to that of the TWs: the mean thickness of the 10kDa gels was 9.56 ± 0.86 microns and the mean thickness of the 20kDa gels was 12.59 ± 1.03 microns.

A modified ball drop test was used as a 3-point flexural tensile evaluation to determine the Young’s moduli of the porated hydrogels in their hydrated state [12]. The mean Young’s modulus of the 10kDa hydrogels was 83.8 ± 9.75kPa and the mean Young’s modulus of the 20kDa hydrogel was 54.7 ± 4.98kPa (Fig 1D). Polycarbonate, polystyrene, and glass have reported Young’s moduli of over 2GPa [12,18–20], which is a marked contrast to soft tissue, which ranges from 2 to 80kPa [17].

### Ultrathin porated hydrogels support endothelial cell monolayers and maintenance of venular phenotypes

**Fig 2:**
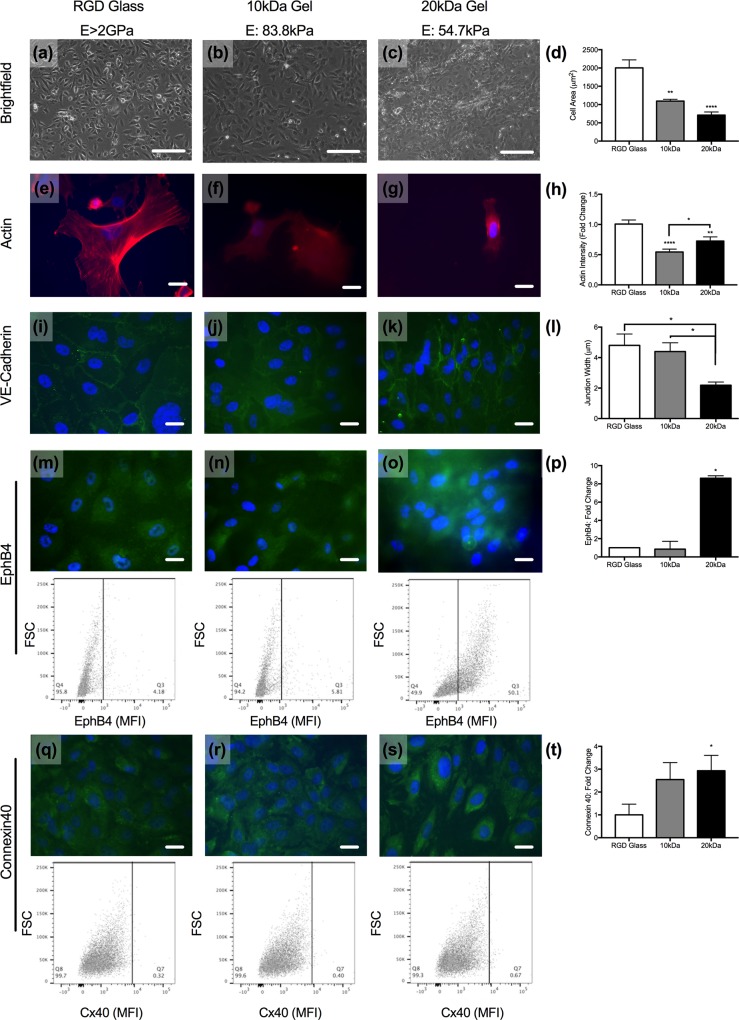
EC phenotype is modulated by substrate stiffness. (a-c) Brightfield images of ECs cultured on RGD-coated glass (E>2GPa), 10kDa (E: 83.8kPa), or 20kDa (E: 54.7kPa) hydrogels. Scale bars are 80 microns. (d) Quantification of cell size across all three substrates. **p<0.01, ****p<0.0001 as determined by one-way ANOVA. (e-h) Phalloidin (red) and DAPI (blue) staining on sub-confluent ECs on glass (e), 10kDa (f), and 20kDa (g) gels and quantification of the actin intensity (h). *p<0.05, **p<0.01, ****p<0.0001 as determined by unpaired t-test. Intensity of 10kDa and 20kDa images augmented to increase staining visibility. (i-l) VE-cadherin (green) and DAPI (blue) staining on ECs on glass (i), 10kDa (j), and 20kDa (k) gels and quantification of the junctional width (l); *p<0.05 as determined by one-way ANOVA. (m-o) EphB4 (green) and DAPI (blue) staining on ECs on glass (m), 10kDa (n), and 20kDa (o) gels with representative flow cytometry dot plots shown below each image. Quantification of the intensity of expression (p); *p<0.05 as determined by unpaired t-test. (q-s) Connexin40 (green) and DAPI (blue) staining on ECs on glass (q), 10kDa (r), and 20kDa (s) gels with representative flow cytometry dot plots shown below each image. Quantification of the stain intensity by flow cytometry; *p<0.05 by unpaired t-test (t). Scale bars in (e-s) are ten microns.

**Fig 3:**
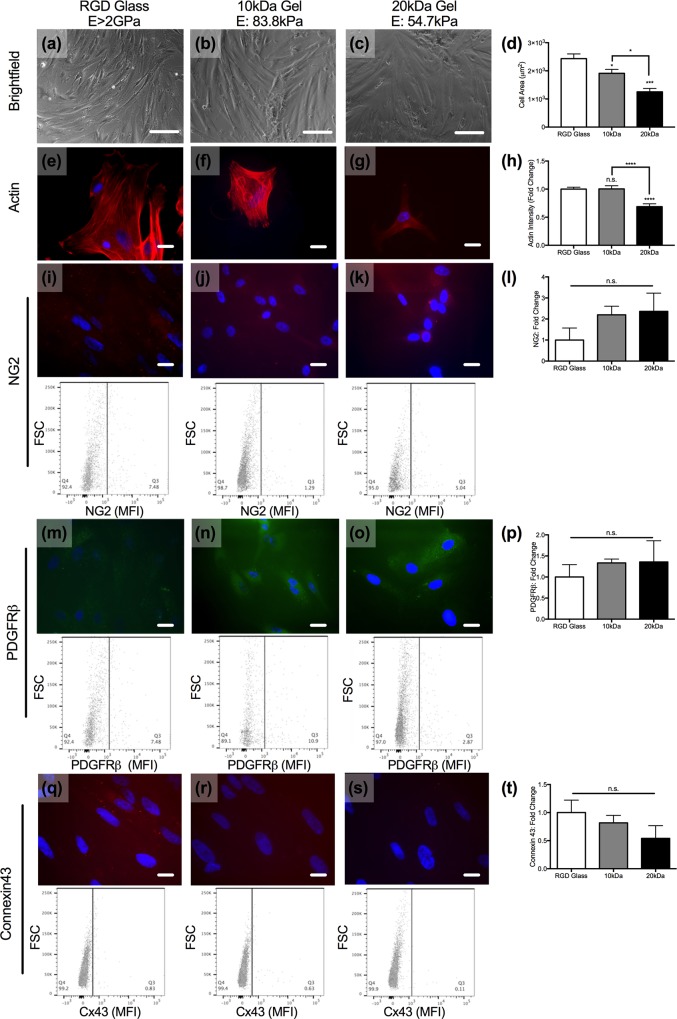
PC phenotype on glass and PEG hydrogels. (a-c) Brightfield images of PCs cultured on RGD-coated glass (E>2GPa), 10kDa (E: 83.8kPa), or 20kDa (E: 54.7kPa) hydrogels. Scale bars are 80 microns. (d) Quantification of cell size across all three substrates. *p<0.05, ***p<0.001 as determined by one-way ANOVA. (e-h) Phalloidin (red) and DAPI (blue) staining on sub-confluent PCs on glass (e), 10kDa (f), and 20kDa (g) gels and quantification of the actin intensity (h). ****p<0.0001 as determined by unpaired t-test. (i-l) NG2 (red) and DAPI (blue) staining on PCs on glass (i), 10kDa (j), and 20kDa (k) gels with representative flow cytometry dot plots shown below each image. Quantification of intensity by flow cytometry (l). (m-o) PDGFRβ (green) and DAPI (blue) staining on PCs on glass (m), 10kDa (n), and 20kDa (o) gels with representative flow cytometry dot plots shown below each image. Quantification of the intensity of expression (p). (q-s) Connexin43 (green) and DAPI (blue) staining on PCs on glass (q), 10kDa (r), and 20kDa (s) gels with representative flow cytometry dot plots shown below each image. Quantification of staining intensity by flow cytometry (t). Scale bars in fluorescent images are 10 microns.

Microvascular ECs and PCs reside on or within flexible BMs. The majority of studies that investigate the cell-cell signals that facilitate microvascular pro-inflammatory behavior have been conducted on rigid glass and TWs. Replacing traditional culture substrates with elastic membranes provides more physiologically-relevant insight into microvascular health and vascular stiffening in disease. The ability of porated PEG hydrogels to support vascular cell growth was confirmed by demonstrating that ECs and PCs were capable of adhering to and forming monolayers. Bio-inert PEG scaffolds were rendered bioactive through presentation of the small tri-peptide Arg-Gly-Asp (RGD) throughout the structure. RGD-coated (20%) glass was used as a control for imaging studies, as opposed to opaque TWs. Brightfield microscopy images confirm that all three substrates support the growth of confluent EC and PC monolayers (Fig 2A–2C; Fig 3A–3C).

Substrate rigidity is known to impact cell size [25]. While ECs form confluent monolayers on all three substrates, the individual cell area is reduced as a function of scaffold mechanics (Fig 2D). Prior research has demonstrated that microvascular ECs range in area from 300 to 1200μm [26]. ECs cultured on glass had the largest surface area; the mean EC area was 2003 ± 217 μm^2^. In contrast, the mean EC area of ECs cultured on both the 10kDa (E: 83.8kPa) and the 20kDa (E: 54.7kPa) hydrogels fell within the reported value of ECs in the microvasculature. The mean EC surface area on the 10kDa and 20kDa hydrogels were 1094 ± 48.3 μm^2^, and 711 ± 84.4 μm^2^. Further confirming the role of mechanics on cell size, we evaluated cytoskeletal protein crosslinking in the cells on the three scaffolds (Fig 2E–2H). Quantification of phalloidin-labeled ECs demonstrated that the mean actin intensity per cell was significantly higher on RGD-coated glass compared to on the two hydrogels (Fig 2H). Actin intensity of ECs cultured on the 20kDa hydrogels (E: 54.7kPa) was slightly higher than the intensity of ECs on 10kDa hydrogels (E: 83.8kPa), though staining intensity normalized by cell area and size suggests no significant difference in actin intensity of ECs cultured on either hydrogel (S1A Fig).

Immunofluorescence staining of VE-cadherin junctions on ECs across all three substrates confirmed that the EC monolayers formed are functionally integrated; VE-cadherin was present and appropriately localized to intercellular boarders on all three substrates (Fig 2I–2K). However, measurements of the junctional widths demonstrated that the width of VE-cadherin junctions is a function to the substrate stiffness (Fig 2I). ECs cultured on RGD-coated glass had the widest VE-cadherin junctions (4.8 ± 0.75μm) compared to ECs on 10kDa or 20kDa hydrogels. Reducing the substrate stiffness decreased the width of the VE-cadherin junctions to 4.4 ± 0.57μm and 2.2 ± 0.21μm on the 10kDa (E: 83.8kPa) and 20kDa hydrogels (E: 54.7kPa), respectively.

Basement membrane stiffness can contribute to EC heterogeneity throughout the vascular tree [27]. We sought to determine if ECs cultured on a scaffold that more closely mimics the BM mechanics would adopt a venular phenotype. Immunofluorescence imaging and flow cytometry confirmed that EphB4, a marker of venous ECs [9], was expressed on all ECs tested (Fig 2M–2P). Flow cytometry quantification demonstrated that ECs cultured on the RGD-coated glass (E>2GPa) and 10kDa hydrogels (E: 83.8kPa) expressed comparable levels of EphB4 (Fig 2M and 2N; representative dot plots). ECs cultured on 20kDa hydrogels (E: 54.7kPa), however, expressed approximately 8-times the amount of EphB4 as those cultured on glass (Fig 2O and 2P). Thus, ECs cultured on the 20kDa hydrogel adopted a phenotype that is more venular than the ECs on stiffer substrates.

*In vivo*, connexin40 expression is found throughout the vascular tree, and the ability to express connexin40 in culture is a measure of EC health and functionality [28]. Flow cytometry and immunofluorescence imaging validate that connexin40 expression is retained on ECs cultured on both the RGD glass and the hydrogels (Fig 2Q–2T). Connexin40 expression on ECs increased as substrate elasticity decreased. ECs cultured on the 10kDa hydrogel (E: 83.8kPa) expressed 2.55-times more connexin40 than ECs on RGD-glass (E<2GPa; p = 0.13), and connexin40 expression was 2.93-fold greater (p<0.05) on ECs on the 20kDa hydrogel (E: 54.7kPa) compared to ECs on glass. The expression of connexin43, another common gap junction protein within EC gap junctions, is often increased during vascular injury. We confirm that there is no change in connexin43 expression on ECs across the three substrates tested here (S2 Fig).

### Ultrathin porated hydrogels support pericyte monolayers and maintenance of venular phenotypes

As did ECs, PCs adhered to and grew on all three substrates, and their size was substrate-dependent (Fig 3A–3D). The surface area of PCs decreased as the stiffness of the scaffold decreased; PCs on 10kDa hydrogels (E: 83.8kPa) were approximately 80% of the size of PCs on glass (E>2GPa; 1912 ±m^2^ v. 2433 μm^2^) and PCs on 20kDa hydrogels (E: 54.7kPa) were approximately 55% the size of those on glass (1255 v. 2443 μm^2^). Analysis of the PC actin intensity revealed that, as with ECs, there was a significant decrease in actin intensity associated with the 20kDa gels relative to the other scaffolds; actin intensity on the 20kDa gels (E: 54.7kPa) was approximately 70% of the mean values associated with PCs cultured on RGD-coated glass. In contrast to ECs, actin intensity of PCs on 10kDa hydrogels (E: 83.8kPa) was comparable to PCs on RGD-coated glass (Fig 3E–3H).

We fluorescently labeled PCs for NG2 and platelet derived growth factor receptor beta (PDGFRβ), two of the common markers of vascular PCs. PCs on all three substrates stained positive for these two markers, with no significant difference in their expression across glass or hydrogels as determined by fluorescence imaging and flow cytometry (Fig 3I–3P). Vascular mural cells, including PCs, utilize connexin43 to interact with EC connexins [29]; flow cytometry reveals that connexin43 is present on PCs cultured on all three scaffolds, with no significant change in its expression across the three PC populations (Fig 3Q–3T). Overall, both ECs and PCs demonstrated stiffness-associated differences in actin intensity, but only ECs showed differences in markers that indicate vascular cell phenotype or vascular stability.

### ECs and PCs on hydrogels respond to pro-inflammatory signals

**Fig 4:**
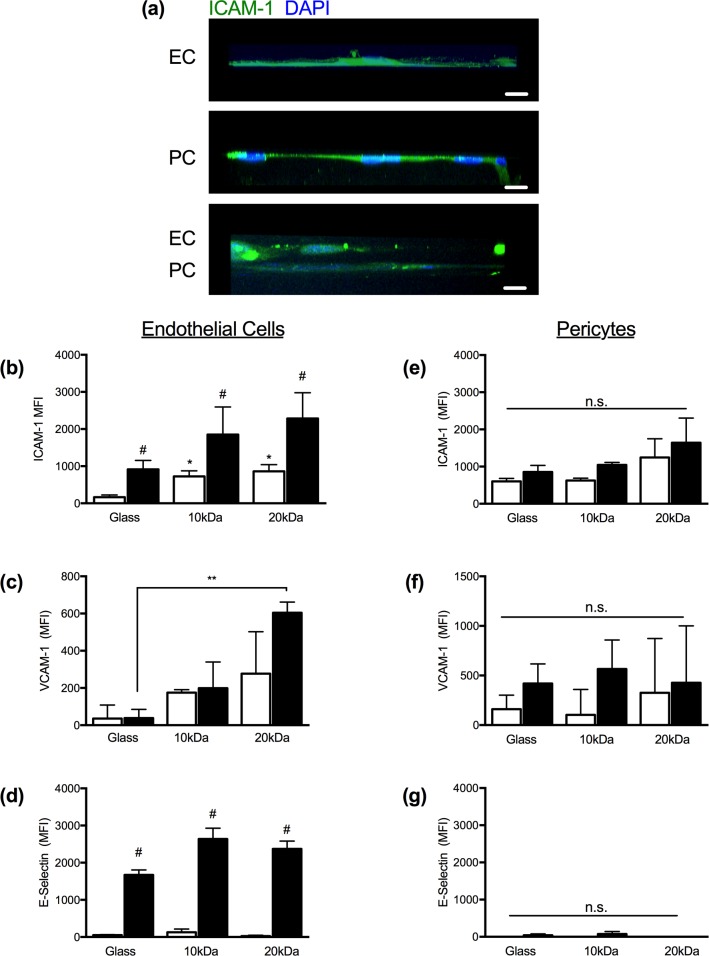
Vascular cell response to TNFα-activation on porated PEG gels. (a) EC (top), PC (middle) and EC/PC (bottom) cell layers in profile stained for ICAM-1 (green) and DAPI (blue) on 20kDa gels. Scale bars are ten microns. (b-d) Flow cytometry results of ECs stained for ICAM-1 (b), VCAM-1 (c), and E-selectin (d) under control and TNFα-activated conditions on RGD-coated glass (E>2GPa), 10kDa (E: 83.8kPa), or 20kDa (E: 54.7kPa) hydrogels. (e-g) Flow cytometry of PCs stained for ICAM-1 (b), VCAM-1 (c), and E-selectin (d) under control and TNFα-activated conditions on RGD-coated glass (E>2GPa), 10kDa (E: 83.8kPa), or 20kDa (E: 54.7kPa) hydrogels.

Vascular stiffening is associated with increased inflammation [30]. However, the extent to which BM stiffening alters the pro-inflammatory phenotype of ECs and PCs is less well established. We, therefore, evaluated EC and PC expression of intercellular adhesion molecule (ICAM)-1, vascular cell adhesion molecule (VCAM)-1, and E-selectin across all three substrates. ICAM-1 (Fig 4A and 4B) and VCAM-1 (Fig 4C) expression on ECs was sensitive to changes in the substrate stiffness. Under quiescent conditions, ICAM-1 expression on ECs cultured on 10 and 20kDa hydrogels was significantly higher than those cultured on glass under non-activated conditions; the mean fluorescence intensities (MFIs) of ECs cultured on glass, 10kDa (E: 83.8kPa) and 20kDa (E: 54.7kPa) hydrogels were 164, 724, and 862, respectively. TNFα-activation induced a significant increase in ICAM-1 expression on ECs across all substrates. Although ICAM-1 MFIs remained elevated on ECs cultured on hydrogels (10kDa: 1851; 20kDa: 2254) relative to ECs on glass (MFI: 918), these differences were not statistically significant.

EC expression of VCAM-1 was statistically equivalent under control conditions across all three substrates. Following activation with TNFα, EC VCAM-1 expression was 5-fold higher on ECs cultured on 10kDa hydrogels (E: 83.8kPa) and 15-fold higher (**p<0.01) on ECs cultured on the 20kDa hydrogel (E: 54.7kPa) compared to their counterparts on the RGD-coated glass (E>2GPa). In contrast, EC presentation of E-selectin under both non-activated and TNFα-activated conditions was independent of substrate stiffness (Fig 4D).

The response of PCs to TNFα-activation (4hrs) has previously been demonstrated to be less robust than that of ECs [31]. Here, as determined by flow cytometry, PCs showed modest increases in ICAM-1 following activation with TNFα, but this increase was only statistically significant in the case of the 10kDa hydrogel. ICAM-1 expression under both non-activated and activated conditions, however, was not influenced by the substrate mechanics and there was no significant difference between the three populations of control or TNFα-activated ECs. Similarly, PC expression of VCAM-1 did not differ across the substrates tested here (Fig 4F). As negative control, we evaluated the PC expression of E-selectin, which, as expected, was absent (Fig 4G). As demonstrated under quiescent conditions, the phenotype of ECs under inflammatory conditions was significantly altered by differences in the mechanical environment, whereas PCs were largely unaffected by substrate mechanics.

### Porated PEG hydrogels can support complex multi-cellular events of the leukocyte adhesion cascade

During inflammation, neutrophils traverse the thin postcapillary venule wall (S3 Fig). We sought, then, to reconstruct the complex microvascular structure, inclusive of EC, PC and BM mimetic scaffold to determine whether the complex structure could support neutrophil migration across PEG supported EC and PC monolayers, and EC/PC bilayers.

Under non-activated and TNFα-activated (4hrs) conditions we observed an atypically low efficiency of neutrophil transmigration across the hydrogels as compared to standard Boyden chamber assays (S4 Fig). We fixed and examined the scaffolds to determine where neutrophils were being captured during their migration across ECs, PCs, and BM-like scaffolds. With this method we did observe an increase in neutrophil accumulation in the space between EC and PC, directly in contact with the hydrogels.

**Fig 5:**
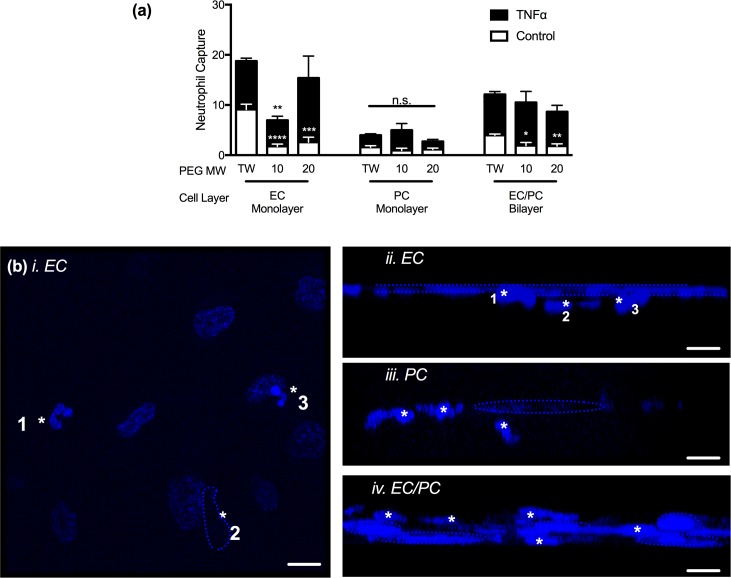
Neutrophil capture on with EC or PC monolayers or EC/PC bilayers. (a) Quantification of neutrophil capture within EC monolayers, PC monolayers, or EC/PC bilayers on transwells (TWs), 10kDa hydrogels, or 20kDa hydrogels under quiescent or TNFα-activated conditions. *p<0.05, **p<0.01, ***p<0.001, and ****p<0.0001 when compared to TWs under the same culture conditions. Unless noted, changes between scaffolds are not significant. (b) Confocal microscopy results of neutrophils interacting with cellular layers on 20kDa hydrogel scaffolds. *i and ii*. Projection (i; * indicates three distinct neutrophils) and profile (ii; *neutrophils, dashed line outlines EC nuclei layer) of an EC monolayer with neutrophils. *iii*. Profile of PCs (dashed line outlines PC nucleus) and neutrophils (*denotes neutrophils). *iv*. EC/PC bilayer (apical layer: EC; basal layer: PC; dashed line outlines cell nuclei) and neutrophils (*denotes neutrophils). Scale bars are 10 microns.

In quiescent, non-activated EC monolayers, neutrophil capture was greatest on TWs and significantly reduced when EC were cultured on 20kDa hydrogels (28% of neutrophil capture on TWs; p<0.001) and 10kDa hydrogels (19.2% of neutrophil capture on TWs; p<0.0001; Fig 5A and 5B). Following TNFα-activation, neutrophil capture on EC monolayer models was statistically equivalent on both TWs and 20kDa hydrogels, though neutrophil capture on the 10kDa hydrogel remained below that of the TW (**p<0.01).

Under non-activated conditions, the number of neutrophils captured within the PC monolayer and scaffolds (Fig 5A and 5B-iii) was relatively low compared to neutrophil capture by EC monolayers. There was not a statistically significant impact on neutrophil capture under TNFα-activated conditions. However, within the EC/PC bilayer, neutrophil capture in the non-activated EC/PC bilayers followed trends similar to those seen with the EC monolayer (Fig 5A and 5B-iv); neutrophil capture was significantly greater on the TWs compared to 10kDa (p<0.05) and 20kDa (p<0.01) hydrogels. Though, following TNFα-activation, the EC/PC bilayer on the hydrogels supported neutrophil capture that was not statistically differing from capture in the TWs. Together these data suggest that quiescent EC monolayers and EC/PC bilayers on TWs facilitate higher levels of neutrophil capture than do EC and EC/PC cultures on elastic membranes, yet, following TNFα activation, these differences are equilibrated.

### Porated PEG hydrogels support neutrophil chemotaxis, while RGD also activates neutrophils

**Fig 6:**
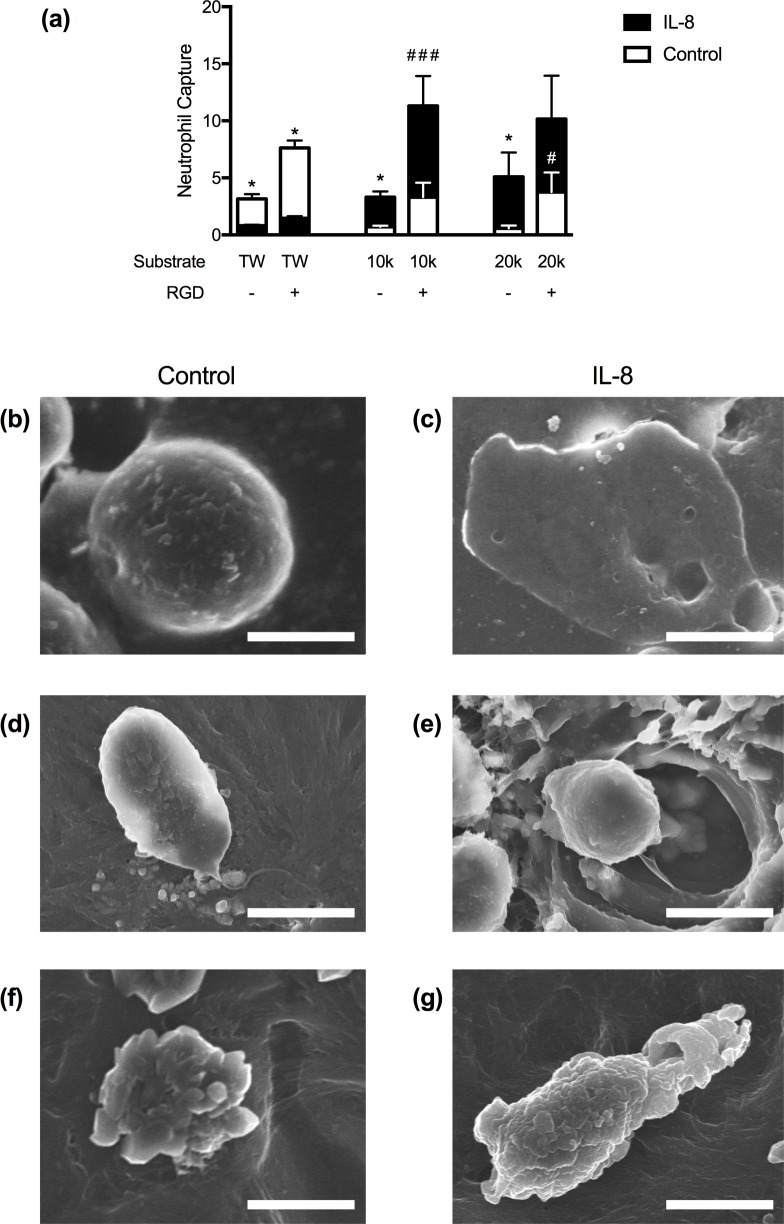
Neutrophil interactions with porated PEG hydrogels. (a) Neutrophil capture in transwells (TWs) and hydrogels without or with RGD. *p<0.05 for control v. IL-8 on the same scaffold, #p<0.05, ###p<0.001 for comparisons between the same condition on scaffolds +/- RGD. All statistical differences determined by a two-way ANOVA. Changes are not significant unless otherwise denoted. (b-g) SEM micrographs of human neutrophils on TWs (b-c), 10kDa porated gels (d-e), or 20kDa gels (f-g) under control (left) or IL-8 (right) conditions. Scale bars are 5 microns.

Both basement membrane mechanics and composition may facilitate differences in leukocyte recruitment across microvascular structures. Having observed that neutrophils could undergo transendothelial migration, but remained captured in the post-EC space, we evaluated the extent to which basement membrane bioactivity was responsible for reduced transvascular migration efficiency. RGD, an integrin-binding motif included to enable EC and PC adhesion to the hydrogels, also promotes neutrophil adhesion [32]. Thus, we compared neutrophil capture on all three substrates in the absence or presence of RGD (Fig 6).

Neutrophil capture increased by 2.6, 4.7-, and 7-fold on TWs, 10kDa, and 20kDa hydrogels, respectively, containing RGD, as compared to those that did not (Fig 6A). SEM imaging revealed that neutrophils were morphologically distinct and activated on RGD-containing scaffolds. As compared to a quiescent and spherical neutrophil on the TW (Fig 6B), Fig 6D shows a representative neutrophil on the 10kDa gel with a long tether extending from the cell body and Fig 6F shows a neutrophil that exhibits membrane ruffling indicative of cell activation. Quantification of neutrophil aspect ratio on the hydrogels in the absence and presence of RGD confirms RGD does increase neutrophil polarization, a metric of neutrophil activation (S5B Fig).

In contrast to haptotaxis, an IL-8 gradient facilitates directional migration and resulted in a 7.8-fold increase in the number of cells that migrated through the TWs (S5A Fig). SEM imaging demonstrated that neutrophils on RGD-coated TWs undergo dramatic changes in cell morphology following the addition of IL-8. Neutrophils on TWs transformed from spherical cells to highly spread and flattened cells in response to IL-8 (Fig 6B and 6C). Overall, neutrophil interactions on the TWs appear to take a binary form of either quiescent or highly activated, rather than facilitating the range of morphological changes that are possible for neutrophils during migration [7,33]. This corroborates previous studies that indicate that neutrophil spreading can be greatest on stiffer substrates [7].

The creation of a chemotactic IL-8 gradient further activated the neutrophils and increases the neutrophil capture within the gel. Neutrophil capture was greatest on 10kDa gels containing RGD, followed by 20kDa gels with RGD. SEM imaging demonstrates that the neutrophils within the gels have undergone a more polarized morphology relative to the control conditions and are utilizing the pore network within the hydrogels to navigate towards the IL-8, the rate of migration in a bioadhesive and elastic scaffold is less efficient than that on the stiffer TW (Fig 6E and 6G).

## Discussion

Cellular phenotype and function is dynamic and often based on highly localized mechanical and biological cues. Throughout the vascular tree, local mechanical and biological cues differ; as a result, resident ECs and PCs adopt heterogeneous morphologies and functions. Unfortunately, many of the localized biological and mechanicals signals are lost in *in vitro* models. Commercially available culture substrates like polystyrene, glass, and TWs are ubiquitously used, but fail to replicate the elasticity found in native blood vessels and lack the complex signaling moieties that are found within the BM. Results obtained from these models have yielded countless insights into the mechanisms that govern health and disease, yet, the ability to apply or translate these insights may be hindered as a result of incomplete replication of *in vivo* conditions. Here, we present a simple and novel method for generating thin, porated PEG hydrogels that provide an alternative to stiff and/or bio-inert *in vitro* models of biological membranes.

Unlike traditional culture substrates (e.g. glass, polystyrene, or polycarbonate) that are often inelastic and bio-inert, the human BM is a soft, elastic, and biologically-active sheet that provides local mechanical and biological cues to cells. By using 10kDa and 20kDa PEG, we are able to create elastic membranes with average Young’s moduli of 83.8kPa and 54.7kPa, falling within the range of human BM (E: 2-80kPa). Finally, we incorporated RGD three-dimensionally throughout the hydrogels presenting bioactive motifs ubiquitously presented in the BM. The combination of biomimetic architecture, mechanics and bioactivity within these hydrogels leads us to conclude that PEG hydrogels more accurately replicate human BMs than traditional experimental substrates. The modularity and tunability of this system opens the door to match specific physiological systems or disease models.

Porated PEG hydrogels support EC and PC growth and culture, allowing us to replicate the composite structure of the postcapillary venule wall and to evaluate leukocyte recruitment. Notably, ECs respond remarkably to reductions in the scaffold elasticity by decreasing cell area, increasing venular phenotype, increasing expression of intercellular exchange molecules. The surface area of ECs was reduced from by half when cells were cultured on porated hydrogels, as opposed to glass, to adopt a cell surface area that falls within the range reported for ECs *in vivo* (300–1200 μm^2^)[27]. While ECs do connexin43, connexin40, EphB4 and VE-cadherin on glass, culture on elastic hydrogels increased the expression of connexin 40, EphB4 and induced vascular junction tightening by VE-cadherin. ECs cultured on the 20kDa hydrogel had a higher expression level of EphB4, a marker of venous cells, than those cultured on glass. In contrast, connexin40 was lower on glass than on elastic hydrogels. Down-regulation of connexin40 is often associated with the pathological dysfunction of ECs [34]. Both the relative reduction of cell size and the relative increase in EphB4 and connexin40 of ECs cultured on 20kDa hydrogel suggest that elastic BM mimics (E: 54.7kPa) yield an EC phenotype similar to one found in healthy venules *in vivo*.

In our composite system of the microvasculature structure, neutrophil capture was dictated by both the presence of resident vascular cells and the BM features. Quiescent cell expression of EC ICAM-1 and VCAM-1 was higher on elastic hydrogels than on glass, though TNFα-activation facilitated statistically equivalent expression of ICAM-1 on ECs regardless of the scaffold. Typically considered a primary mediator of neutrophil capture, high expression of ICAM-1 alone was insufficient to translate to neutrophil capture on EC. Neutrophil capture on EC monolayers was positively correlated with actin intensity per cell, not ICAM-1 intensity; therefore, neutrophil capture was the greatest when EC were cultured on TWs. This builds upon prior studies that suggest that increased substrate stiffness facilitates cytoskeletal remodeling that will promote appropriate presentation of adhesion molecules for efficient inflammatory cell recruitment [8–10].

Even in a quiescent EC/PC bilayer, neutrophil recruitment on hydrogels was lower than was observed on TWs. While, TFNα-activation increased neutrophil capture across EC/PC bilayers on all scaffolds, the increase was the most substantial on the elastic hydrogels; activation of the EC/PC bilayer on the 10kDa hydrogel resulted in 4.5-fold increase in neutrophil capture. *In vivo*, a 10-fold increase in neutrophil recruitment is observed during *in vivo* inflammation following LPS induced injury. While the 10kDa EC/PC bilayer does not fully replicate those *in vivo* observations, it does represent an improvement over the TW.

In contrast to EC, substrate mechanics had little impact on PC phenotype as relates to vascular function through expression of NG2, PDGFRβ, and connexin43. Further, PC responsiveness to TNFα was not modified when substrate mechanics was varied, as PCs did not present differing levels of leukocyte adhesion molecules nor support differing levels of leukocyte transmigration or capture.

In spite of dissimilarities in ICAM-1 and VCAM-1 expression on ECs associated with differences in substrate stiffness, corresponding differences in neutrophil transmigration as traditionally measured were not seen. Rather, our data highlight that the largely overlooked interactions between neutrophils and the BM may play a large role in determining transvascular migratory efficiency. Although both the EC monolayer and the EC/PC bilayer captured relatively large number of neutrophils following TNFα-activation, post-EC migration was restricted by the BM. Comparing neutrophil interactions with the three BM-like scaffolds evaluated here demonstrates that the bioactivity and physical properties of the BM may limit neutrophil migration and delays the neutrophils exit into the vascular space.

Overall, we present a straightforward method for creating thin, porated, elastic, and bioactive PEG hydrogels as a method to replicate human BMs *in vitro*. These hydrogels are capable of supporting monoculture, co-culture, and cellular migration assays. While demonstrating the utility of this system as an *in vitro* model of the human postcapillary venule, these scaffolds can be altered structurally, mechanically, and biologically, thereby having the potential to replicate various BM inclusive structures to yield insight into the ability of BM to modulate cell behavior.

## Supporting information

S1 Fig Vascular cell actin organization on hydrogels. Actin integrated intensities for ECs (a) and PCs (b) cultured on RGD-coated glass, 10kDa, or 20kDa hydrogels. ***p<0.001; ****p<0.0001 as determined by unpaired t-tests.
(TIFF)

S2 Fig EC expression of connexin43. EC expression of Cx43 as determined by flow cytometry. There are no statistically significant changes between conditions.
(TIFF)

S3 Fig Eosin staining of human skin and vascular BMs. (a and b) Confocal images of eosin stained human skin biopsies taken from patients with TNFα-mediated inflammation. Arrows denote BM; * denote migrating or perivascular cells. Scale bars at 5μm.
(TIFF)

S4 Fig Neutrophil transmigration through vascular cell-hydrogel scaffolds. Neutrophil transmigrations through EC and PC on hydrogels. Neutrophil transmigration through EC and PC monolayers and EC/PC bilayers on 10kDa and 20kDa hydrogels under non-activated and TNFα-activated vascular cells.
(TIFF)

S5 Fig Neutrophil interactions with BM-like scaffolds. (a) Neutrophil aspect ratios on 10kDa and 20kDa hydrogels in the absence or presence of RGD. *p<0.05 as determined by unpaired t-tests. (b) Neutrophil chemotaxis through TWs and PEG hydrogels under control and IL-8 chemotactic conditions. ****p<0.001 as determined by an unpaired t-test.
(TIFF)
