# Supplementary figures and images for: Biomimetic, ultrathin and elastic hydrogels regulate human neutrophil extravasation across endothelial-pericyte bilayers
Source: PLoS One. 2017 Feb 24;12(2):e0171386. doi: 10.1371/journal.pone.0171386 (PMC5325185; doi:10.1371/journal.pone.0171386)

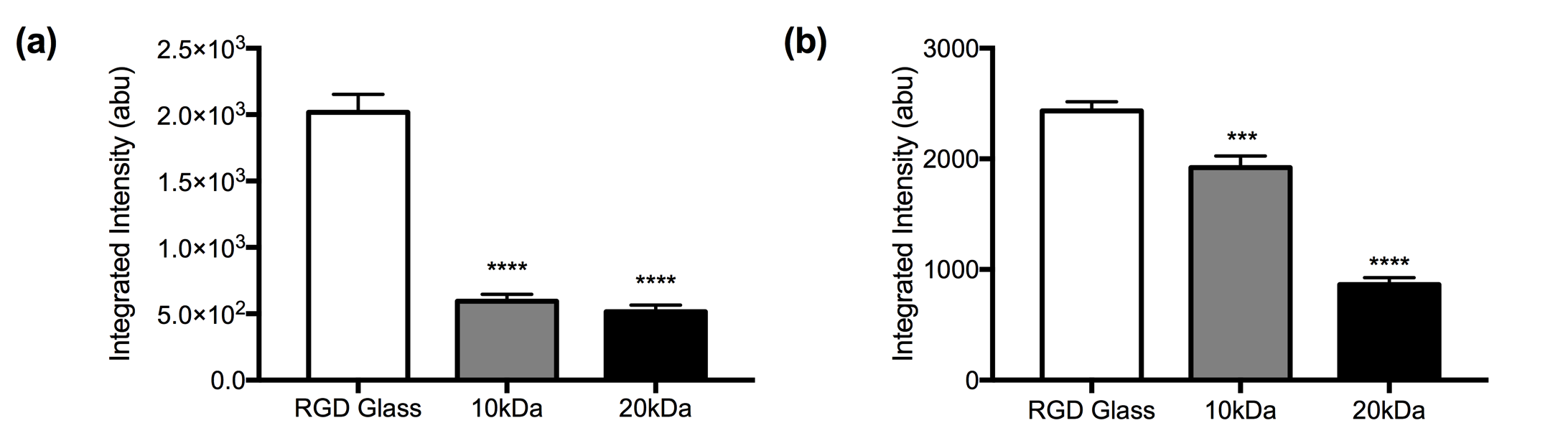

Supplement: S1 Fig — Actin integrated intensities for ECs (a) and PCs (b) cultured on RGD-coated glass, 10kDa, or 20kDa hydrogels. ***p<0.001; ****p<0.0001 as determined by unpaired t-tests. (TIFF) [file pone.0171386.s001.tiff]

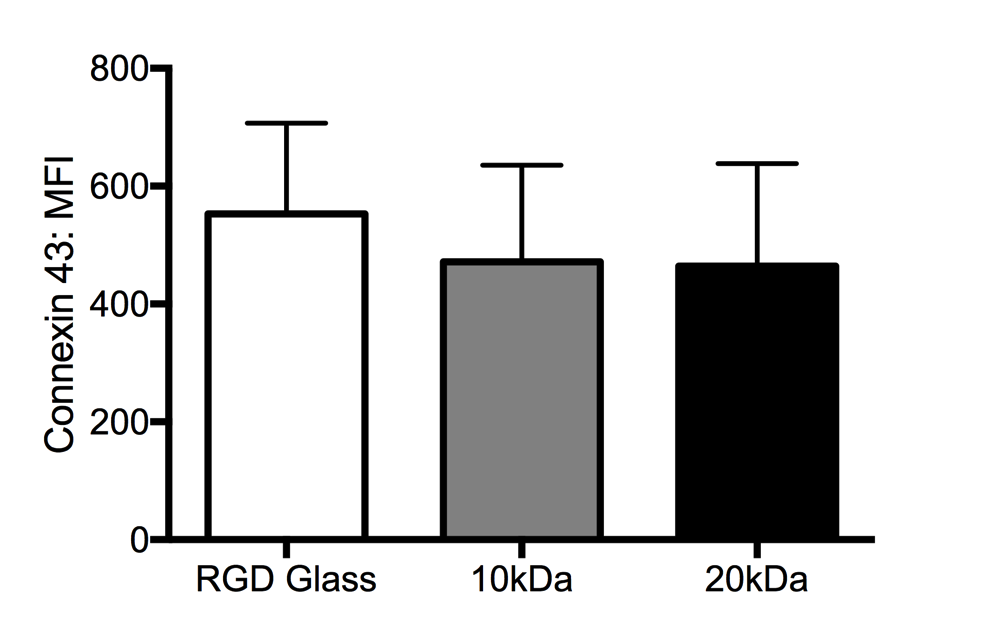

Supplement: S2 Fig — EC expression of Cx43 as determined by flow cytometry. There are no statistically significant changes between conditions. (TIFF) [file pone.0171386.s002.tiff]

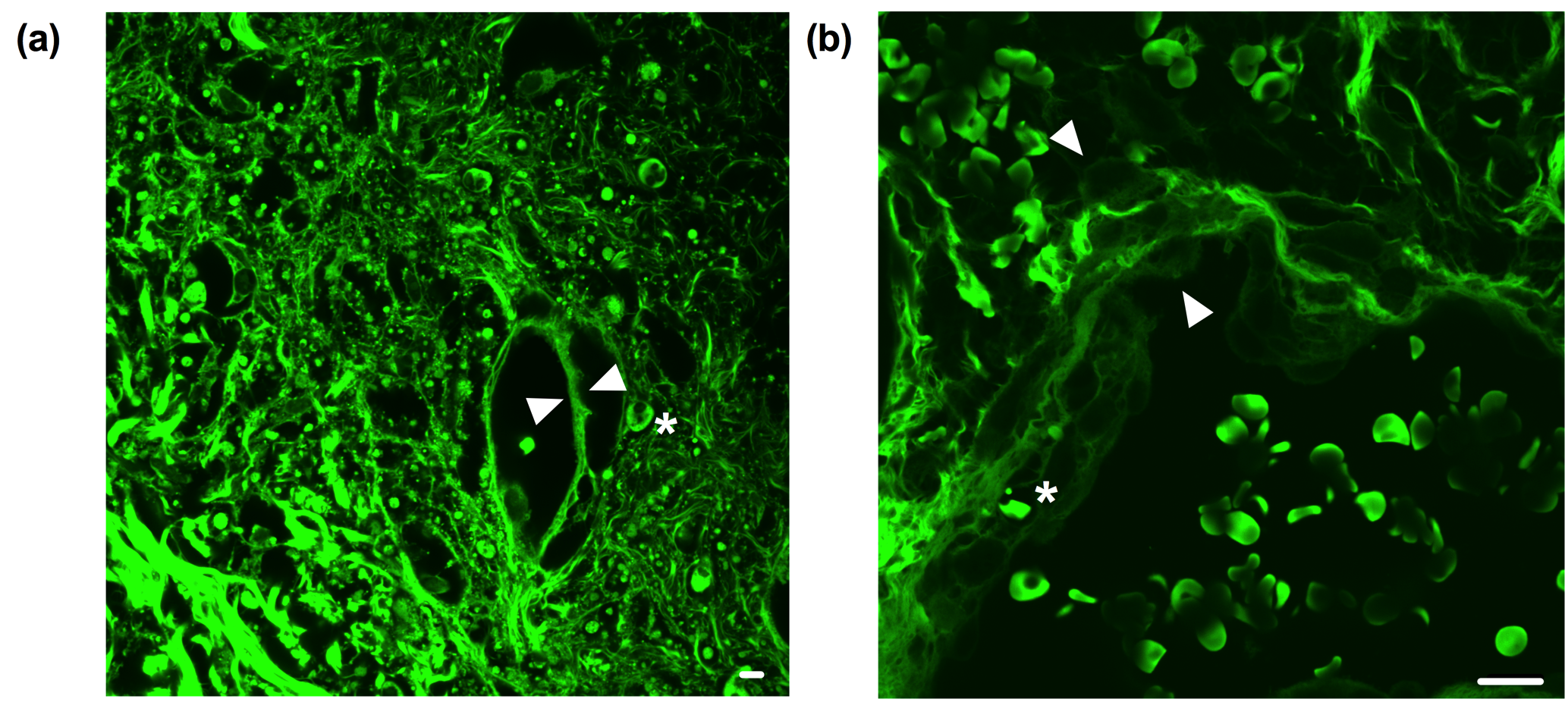

Supplement: S3 Fig — (a and b) Confocal images of eosin stained human skin biopsies taken from patients with TNFα-mediated inflammation. Arrows denote BM; * denote migrating or perivascular cells. Scale bars at 5μm. (TIFF) [file pone.0171386.s003.tiff]

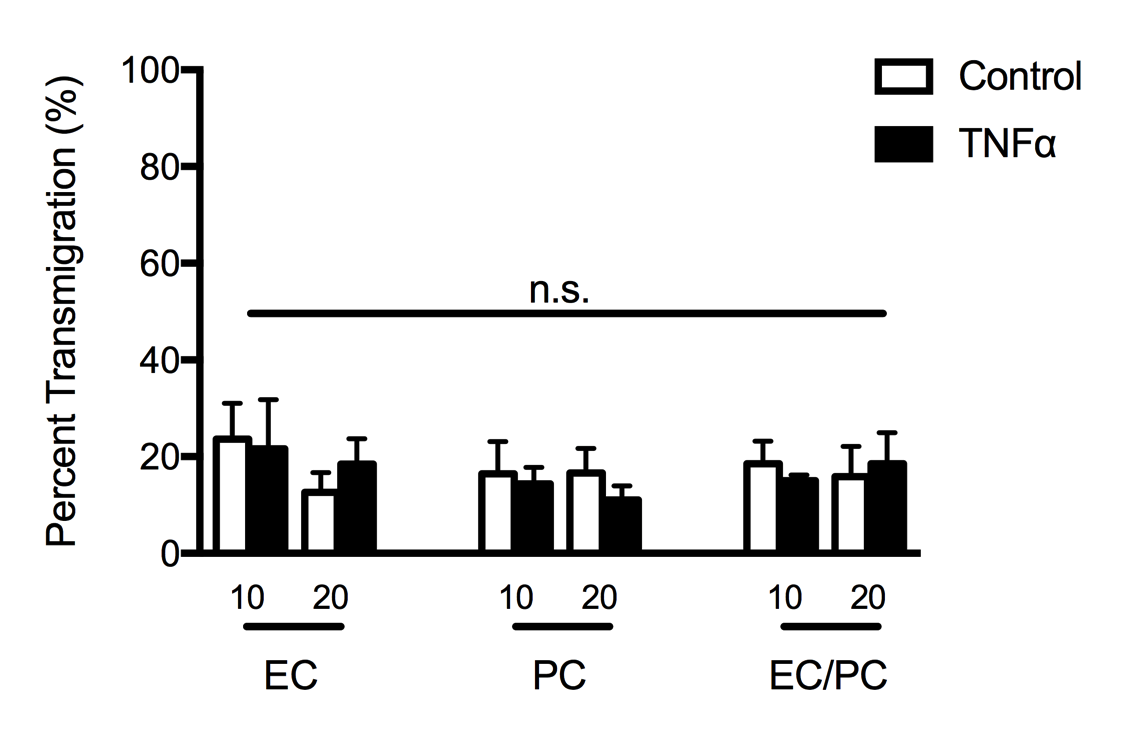

Supplement: S4 Fig — Neutrophil transmigrations through EC and PC on hydrogels. Neutrophil transmigration through EC and PC monolayers and EC/PC bilayers on 10kDa and 20kDa hydrogels under non-activated and TNFα-activated vascular cells. (TIFF) [file pone.0171386.s004.tiff]

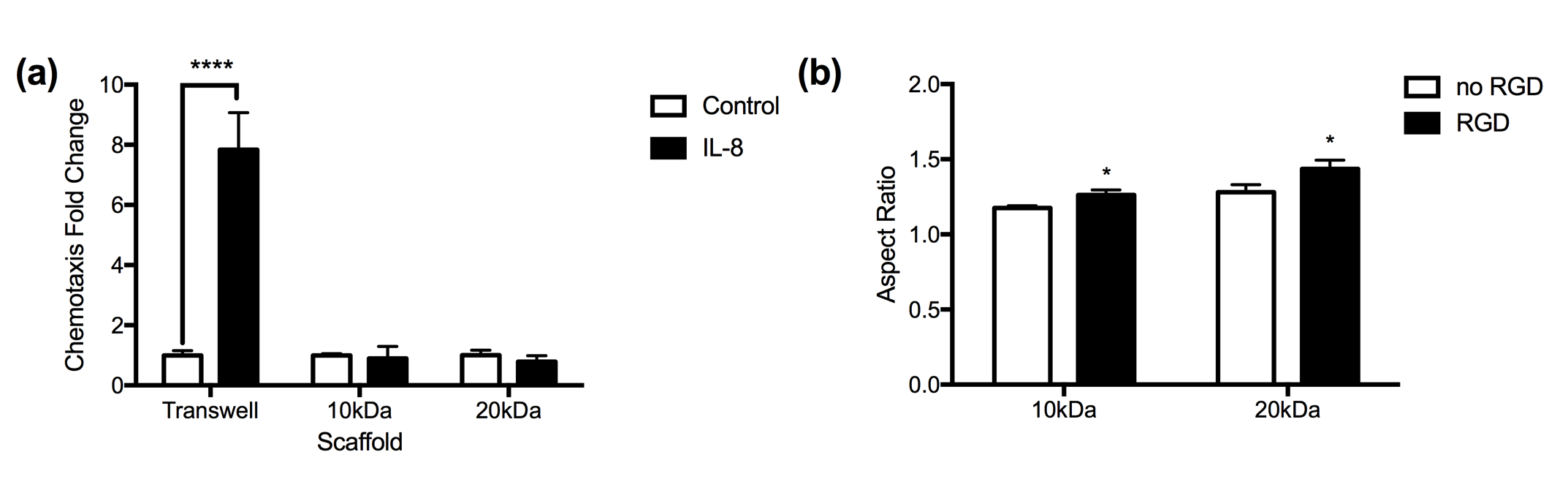

Supplement: S5 Fig — (a) Neutrophil aspect ratios on 10kDa and 20kDa hydrogels in the absence or presence of RGD. *p<0.05 as determined by unpaired t-tests. (b) Neutrophil chemotaxis through TWs and PEG hydrogels under control and IL-8 chemotactic conditions. ****p<0.001 as determined by an unpaired t-test. (TIFF) [file pone.0171386.s005.tiff]
